# Remaining Physiological Barriers in Porcine Kidney Xenotransplantation: Potential Pathways behind Proteinuria as well as Factors Related to Growth Discrepancies following Pig-to-Kidney Xenotransplantation

**DOI:** 10.1155/2018/6413012

**Published:** 2018-03-04

**Authors:** Jigesh A. Shah, Miguel A. Lanaspa, Tatsu Tanabe, Hironosuke Watanabe, Richard J. Johnson, Kazuhiko Yamada

**Affiliations:** ^1^Columbia Center for Translational Immunology, Columbia University Medical Center, New York, NY, USA; ^2^Division of Renal Diseases and Hypertension, University of Colorado, Aurora, CO, USA

## Abstract

Considerable shortages in the supply of available organs continue to plague the field of solid organ transplantation. Despite changes in allocation, as well as the utilization of extended criteria and living donors, the number of patients waiting for organs continues to grow at an alarming pace. Xenotransplantation, cross-species solid organ transplantation, offers one potential solution to this dilemma. Previous extensive research dedicated to this field has allowed for resolution of xenograft failure due to acute rejection, leaving new areas of unresolved challenges as barriers to success in large animal models. Specific to kidney xenotransplantation, recent data seems to indicate that graft compromise can occur due to discrepancies in growth between breeds of donors and significant proteinuria leading to nephrotic syndrome in the recipient. Given these potential limitations, herein, we review potential pathways behind proteinuria, as well as potential causative factors related to growth discrepancies. Control of both of these has the potential to allow xenotransplantation to become clinically applicable in an effort to resolve this organ shortage crisis.

## 1. Introduction

Xenotransplantation remains a promising avenue to address widespread shortages of available organs for transplantation. As a result of extensive previous research, largely in part due to recent advances in genetic engineering, xenografts suitable for human transplantation are on the verge of becoming a clinical reality. The first major breakthrough in the field of xenotransplantation came as a result of the creation of alpha-1,3-galactosyltransferase knockout (GalT-KO) pig donors which were successful in preventing the development of hyperacute xenograft rejection (HAR) [1–3]. More recently, the creation of multitransgenic (Tg) swine donors has allowed investigators to successfully experiment varying immunosuppression regimens consisting of a combination of costimulation blockade, mycophenolate mofetil, and T/B-cell depletion, the effects of which in a cardiac model have allowed for survival of heterotopic hearts in baboons for >2 years [4] and >6 months in a life-supporting kidney model [5, 6]. Despite the improvement in survival from days to months, additional barriers due to antigenic and physiologic differences in cross-species transplantation continue to remain a challenge [7–9].

Specific to challenges limiting xenogeneic kidney transplantation (XKTx), we hypothesize three limiting obstacles: the first is significant posttransplant proteinuria, the second appears to be related to organ growth disparities following xenotransplantation, and the third is the level of immunosuppression needed to control xenogeneic innate and acquired immune responses in an effort to prolong xenograft survival. This review is based predominantly on data from our own studies which focuses on the first two obstacles by reviewing potential pathways behind proteinuria as well as factors related to growth discrepancies. Control of both, along with an adequate control of the xenogeneic immune response, has the potential to allow xenotransplantation to become a clinically applicable solution for addressing the organ shortage crisis.

## 2. Proteinuria and Nephrotic Syndrome Complicating Xenotransplantation

### 2.1. Proteinuria following Pig Kidney Grafts in Nonhuman Primates

Significant proteinuria has been previously reported [9–12] following *α*-1,2-fucosyltransferase pig-to-cynomolgus, as well as following GalT-KO and GalT-KO/hCD39/CD55/CD59 pig-to-baboon xenotransplantation [9–12]. Often, the proteinuria is in the nephrotic range and is associated with vascular thrombosis and infections that can limit the survival of the recipient. Histologically, the glomeruli appear normal or show mild mesangial expansion [12]. Although antibody-mediated rejection could lead to proteinuria [13], we have found that nephrotic syndrome can occur in the absence of any elicited antibody development or complement activation (i.e., antibody-mediated rejection) after pig-to-baboon xenotransplant [9, 12].

While nephrotic syndrome is a common complication following xenotransplantation, investigators at Emory have published data demonstrating low preformed natural antibodies and >133-day survival with minimal proteinuria and absence of serum hypoalbuminemia in rhesus monkeys that have received GalT-KO/hDAF (human decay-accelerating factor) kidneys [5]. Despite these encouraging findings, Pintore et al. continue to report challenges that occur with posttransplant proteinuria and the presence of low molecular weight proteins that are consistently found in recipient urine samples after kidney xenotransplantation in hDAF or multi-Tg GalT-KO pig-to-cynomologus monkey models [10, 11]. In addition to these findings, our lab has extensive experience with hDAF/GalT-KO pig-to-cynomolgus monkey XKTx using an anti-CD40L-based regimen without vascularized thymic grafts, and these recipients commonly developed proteinuria similar to that observed in baboon recipients of GalT-KO pig kidneys (Yamada et al., manuscript in preparation). Given the discrepancy in these results, it is not yet concluded whether specific recipient strains are involved in the absence of proteinuria that is observed in the GalT-KO/hDAF rhesus macaque model [5]. Efforts to resolve proteinuria remain a major focus for the success of XKTx.

#### 2.1.1. Potential Causes for Posttransplant Proteinuria

Our laboratory has previously published data demonstrating that the cotransplantation of vascularized thymic grafts with kidneys from the same GalT-KO donor results in prolonged kidney survival in a life-supporting pig-to-baboon model [3, 12]. Recipient baboons in this study demonstrated in vitro evidence of donor-specific tolerance with the development of early baboon thymopoiesis in vascularized pig thymic grafts, suggesting that the recipients were on a path towards the induction of tolerance. The majority of the recipients however developed significant proteinuria as early as postoperative day (POD) 2, despite relatively normal appearing glomeruli and normal renal function [9, 12]. Based upon these results, it was concluded that the development of proteinuria is not initiated by T-cells or as a result of antibody-mediated rejection. Additionally, histologic examination revealed findings that were remarkably similar to the nephrotic condition known as minimal change disease (MCD) that is common in pediatric populations [14, 15]. Furthermore, the development nephrotic syndrome leads to significant anasarca and increases the risk of developing infections, cortical damage, and graft thrombosis due to microangiopathy. It deserves to be mentioned that although microangiopathy and infections are quite frequent complications following xenotransplantation [12, 16], the fact that these complications can result from nephrotic syndrome alone provides additional rationale to identify and treat the causative mechanism(s) [17].

#### 2.1.2. Approaches to Preventing Proteinuria

A recent study reported that the loss of sphingomyelin phosphodiesterase acid-like 3b (SMPDL-3b) in allogeneic human kidney grafts was related to the development of posttransplant proteinuria in patients with focal segmental glomerulosclerosis (FSGS) [18]. In our laboratory, studies of the pig-to-baboon XKTx model have shown that the administration of rituximab in the perioperative period appears to protect SMPDL-3b/sphingomyelinase activity on porcine podocytes, which in turn delays the development of proteinuria [9]. Given that podocytes are one of the primary cells within the glomerulus, we developed a novel technique to study postxenotransplantation proteinuria using pig podocyte cultures followed by histologic confirmation by staining with antinephrin and antipodocin antibodies. As a result of this porcine podocyte culture, our lab was able to discover two critical findings: (i) SMPDL-3b/sphingomyelinase expression on porcine podocytes plays an essential role in initiating proteinuria and (ii) rituximab (anti-CD20 antibody) binds to porcine SMPDL-3b in the glomeruli of the kidney xenografts, thereby preventing damage from circulating baboon preformed antipig natural antibodies or antiporcine soluble factors.

Given our findings from in vitro studies, rituximab was administered to six baboons in the peritransplant period (treatment group) in order to test its effect in vivo, and the treatment was compared with eighteen baboons that underwent GalT-KO thymokidney transplantation without rituximab administration in the peritransplant period (control group). The onset of 2^+^ proteinuria posttransplant was markedly delayed in the treatment group when compared to the control group. Most of the baboons in the control group developed >2^+^ proteinuria within 2 days following transplantation, as compared to the treatment group where the development of 2^+^ proteinuria occurred >12.50 ± 5.54 days posttransplant. To our knowledge, these findings demonstrated for the first time the ability of rituximab to prevent pig podocyte disruption in an SMPDL-3b-dependent manner with subsequent delay in the development of proteinuria following xenogeneic GalT-KO kidney transplantation in nonhuman primates (NHPs) [9]. However, since this effect lasted only two to three weeks, additional treatment strategies are necessary.

#### 2.1.3. The Role of CD80 Upregulation and CTLA4-Ig to Prevent Proteinuria

A significant breakthrough in understanding the pathogenesis of proteinuria occurred with the discovery that podocytes have antigen-presenting functions [19] and can express the dendritic cell receptor CD80 (also known as B7.1) [20, 21]. Recently, urinary levels of CD80 were reported to be extremely high in a patient who developed minimal change-like nephrotic syndrome following allogeneic stem cell transplantation [22]. Moreover, MCD, the most common cause of proteinuria and nephrotic syndrome in children, has been associated with high levels of CD80 in the urine as well as with CD80 expression in glomerular podocytes in renal biopsies [23, 24]. Children with relapsing MCD have been observed to express CD80 on their podocytes (as seen on renal biopsy) and excrete CD80 in their urine, both of which resolve when they enter remission [23, 24]. Serum from relapsing MCD patients has been shown to induce CD80 expression in cultured human podocytes. Additionally, toll-like receptor ligands 3 (polyIC) and 4 (endotoxin (LPS)) in mice induce glomerular CD80 expression and urinary CD80 excretion and cause transient proteinuria [21, 25–27]. CTLA-4, which binds CD80 and inhibits dendritic cell activation [28], resulted in an immediate remission in a child with MCD [29]. Genetic polymorphisms of CTLA-4 have also been associated with the development of MCD [30, 31].

Given that CD80 has also been hypothesized in the development of other glomerular diseases such as FSGS and diabetic nephropathy [32, 33], its specific role in MCD remains controversial, but these observations strongly suggest that podocyte CD80 activation could represent a response of the podocyte to an antigen, allergen, or hypoxic stimulus. We have also found elevated levels of urinary CD80 excretion in a patient presenting with nephrotic syndrome following bone marrow transplant, as well as in a few patients with posttransplant FSGS (unpublished). Urinary CD80 excretion also appears to be higher in MCD than in FSGS or other glomerular diseases [23, 24, 29, 34], and based upon these observations, we and others have hypothesized that proteinuria may involve a two-hit disorder, in which podocyte CD80 is induced by a virus or allergen but then its expression continues due to an impaired CTLA4 response [35–37]. The administration of CTLA4-Ig has previously been reported to be beneficial in patients with posttransplant FSGS who express CD80 in glomeruli [33]. However, these patients also received plasma exchange along with other immunosuppressive agents and the decrease in proteinuria cannot solely be attributed to CTLA-4-Ig administration [38]. Indeed, some FSGS patients experience overwhelming proteinuria despite normal levels of urinary CD80 suggesting that the mechanism of proteinuria in FSGS is not CD80-driven when compared to MCD.

Our group has also recently discovered that the xenograft nephropathy, the nephrotic syndrome associated following xenotransplantation, is also associated with the induction of CD80 expression in podocytes. In particular, we found that the nephrotic syndrome observed in baboon recipients of pig xenografts has (i) increased urinary CD80 excretion that precedes the development of proteinuria (the urinary CD80 appears to be of both baboon and porcine origin), (ii) CD80 was found to be expressed in glomeruli by immunostaining in biopsies of baboons with nephrotic syndrome, and (iii) multiple doses of CTLA4-Ig therapy added on top of an anti-CD40L-based regimen resulted in a marked reduction in proteinuria with significantly improved survival compared to baboons treated with the anti-CD40L-based regimen without the CTLA4-Ig therapy (Yamada et al., manuscript in preparation). By minimizing proteinuria with this modified protocol, we have recently achieved >6 months of stable renal function in baboon recipients of GalT-KO pig thymokidneys without additional gene modifications [8]. This 193-day life-supporting kidney xenograft recipient is thus far the longest known survival of a GalT-KO kidney without additional gene modifications in a baboon. Most notably, the recipient had unresponsive in vitro pig-specific cellular assays and did not develop elicited baboon antipig antibodies.

#### 2.1.4. Recent Progress Using Multitransgene Donors

Although initial studies demonstrated that GalT-KO kidneys without vascularized thymic grafts survived for 34 days in baboons [3], recently published data from the Pittsburgh group has shown that the use of multi-Tg hCD46/hCD55/EPCR/hCD39 GalT-KO pig donors allows for survival of a life-supporting kidney for >6 months in baboons [6]. These results suggest the beneficial effects of multigene editing for prolonging xenograft survival. Despite these promising results, however, similar attempts by other with hCD39/hCD55/hCD59 and *α*1,2-fucosyltransferase GalT-KO pig kidneys in cynomolgus macaques clearly demonstrated the development of proteinuria [10] indicating that the exact responsible genes have yet to be identified.

**Figure 1:**
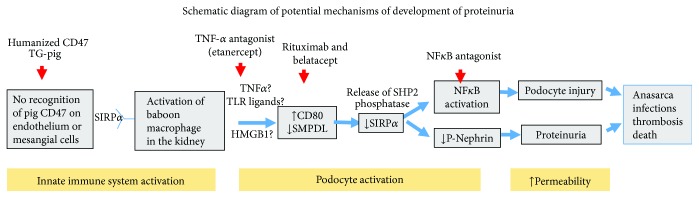
Schematic diagram of potential mechanisms of development of proteinuria. Proposed pathway and causative mechanisms for the development of proteinuria following pig-to-NHP kidney xenotransplantation.

Recently, incompatibilities between porcine CD47 and the baboon signal regulatory protein *α* (SIRP-*α*), an interspecies ligand-receptor responsible for the activation of macrophages and phagocytosis in xenogeneic combinations, have been identified [39–42] (Figure 1). Immune activation of the porcine podocyte leads to expression of CD80 which potentially downregulates SIRP-*α* and SMPDL-3b. The exact role of hCD47 on the development of proteinuria in vitro as well as in vivo using transgenic hCD47-GalT-KO pigs [43] is currently being investigated in our laboratories with preliminary results demonstrating that SIRP-*α* expression is markedly decreased following xenotransplantation of GalT-KO kidneys in baboon that develop proteinuria and that the addition and high expression of hCD47, as well as the addition of hDAF transgenes to GalT-KO pig donors, nearly eliminated proteinuria following thymokidney transplantation in baboons (Yamada et al., manuscript in preparation).

## 3. Differences in Kidney Size May Affect Outcomes in Xenotransplantation

### 3.1. Organ Growth Discrepancies

As the xenotransplant community continues to make steady progress in overcoming immunological barriers, new concerns such as limitations due to growth discrepancies from varying donor strains have risen. Previous work from our laboratory has clearly demonstrated discrepancies in the rate of kidney growth following transplantation utilizing varying donor strains [8]. The importance of matching donors/recipient pairs will become paramount when considering xenotransplantation in special populations, such as pediatric and adolescent recipients, where rapid growth in a limited cavity could cause graft dysfunction. Early attempts to study organ growth following xenotransplantation were led by Soin et al. [44]. The authors have studied 6 cases of pig-to-NHP kidney xenotransplantation where survival was up to 48 days and organs grew for 2 weeks following transplantation, after which point growth plateaued in 3 recipients but continued to grow in the remaining 3 recipients and ultimately led to xenograft failure [44]. While kidneys markedly increased in size due to rejection, the nonrejected xenografts also grow (as judged by weight) at a rate similar to porcine kidneys in their native environment [44]. This natural increase in porcine graft size could lead to a local compression within a limited compartment (a “compartment syndrome” such as observed with the “Page kidney”), especially in pediatric recipients, which could be another barrier to successful xenotransplantation.

To better understand the role of continued growth on organ function and to address the question if organ growth is regulated by intrinsic (organ) factors of extrinsic (host) factors, our laboratory recently investigated the importance of the role of donor strain on organ growth following pig-to-pig kidney and lung allotransplantation and pig-to-baboon kidney xenotransplantation using outbred (Yorkshire) and inbred (MGH miniature swine) donors [8]. Following xenotransplantation, renal volumes were measured at routine intervals at which time was determined that if the ratio of the donor pig kidney volume to recipient body weight was greater than 25 cm^3^/kg, there appeared to be compromise of renal function with eventual graft loss and further alluding to the possibility that growth appeared to be regulated by intrinsic factors. The likely explanation for this deleterious effect is that increased growth causes compromise in renal blood flow to the enlarging xenograft, which in turn leads to cortical ischemia, as the circulating blood volume in the recipient cannot perfuse the graft with enough volume. In addition, the recipient baboons have limited abdominal space that can accommodate a xenograft. As the graft continues to grow, there is eventual development of a compartment syndrome type of effect that further causes decline in renal function as a result of extrinsic compression [8].

In order for xenotransplantation to be clinically applicable, organ growth and size matching will need to be considered, especially in pediatric and petite patients, where continued organ growth from larger donors could cause graft compromise. Although wild type pigs are generally used for production of GalT-KO or multitransgenic GalT-KO pigs because of its productivity, these pigs grow much faster and can reach sizes > 300 kg. In order to more closely examine the morphology of porcine kidneys, Lazo et al. compared anatomical characteristics between 96 Landrace/Yorkshire (used in our previous studies as controls) and 60 Dalland swine which grew to an average of 95 kg and at just 5 months had renal volumes that matched average adult human renal volumes [45]. Despite being anatomically suitable for xenotransplantation, continued growth mismatch of these kidney breeds in human recipients could lead to organ dysfunction if the xenograft continues to undergo unregulated growth as we have previously demonstrated. Miniature swine instead are generally considered the best match for potential human xenotransplantation because of their size, but also because of largely known genetic profile [1, 46–48]. Though early, further research in this field is warranted and currently ongoing to elicit specific factors responsible for continued growth which could be the target of gene therapy [49] in an effort to eliminate this phenomenon.

## 4. Conclusions

The development of xenograft nephropathy following XKTx appears to be closely associated with upregulation of CD80 and loss of SMPDL-3b in porcine podocytes. These changes appear to be due in part to preformed natural antibodies, not elicited antipig antibodies. Additionally, interspecies incompatibilities between CD47 and SIRP-*α* seem to induce glomerular endothelial damages that potentially penetrate the glomerular network and ultimately lead to disruption and damage to porcine podocytes. As work in overcoming immunologic hurdles advances, it appears that additional mechanisms, such as discrepancies in organ growth from donors or varying sizes/strains, may play an important role. Recent work seems to demonstrate that uninhibited growth of transplanted organs may lead to comprise of graft function. As a result of recent data, further work in understanding mechanisms for organ growth, as well as specific size cut-offs, is currently being investigated. Ultimately, as success in controlling posttransplant proteinuria, as well as the selection of appropriate donor strains occurs, XKTx may become a reality, providing patients eagerly waiting on the transplant list will a new avenue of hope.

## Abbreviations

FSGS: Focal segmental glomerulosclerosis
GalT-KO: Alpha-1,3-galactosyltransferase knockout
HAR: Hyperacute rejection
h: Human
hDAF: Human decay-accelerating factor
MCD: Minimal change disease
MHC: Major histocompatibility complex
NHP: Nonhuman primates
PBL: Peripheral blood lymphocytes
POD: Postoperative day
SMPDL-3b: Sphingomyelin phosphodiesterase acid-like 3b
Tg: Transgenic
VTL: Vascularized thymic lobe
XKTx: Xenogeneic kidney transplantation.
